# Identity work among girls with ADHD: struggling with *Me* and *I*, impression management, and social camouflaging in school

**DOI:** 10.3389/fpsyg.2025.1591135

**Published:** 2025-07-16

**Authors:** Jan Grimell, Maja Ericson, Matilda A. Frick

**Affiliations:** ^1^Department of Sociology, Faculty of Social Sciences, Umeå University, Umeå, Sweden; ^2^Department of Psychology, Stockholm University, Stockholm, Sweden; ^3^Child and Adolescent Psychiatry, Department of Medical Sciences, Uppsala University, Uppsala, Sweden

**Keywords:** ADHD, girls, *Me*, *I*, role, identity, impression management, school

## Abstract

The number of individuals diagnosed with ADHD is rapidly increasing in Sweden, with approximately two-thirds of diagnosed children being boys. However, among older adolescents, ADHD now appears to be more common among girls. Despite this, girls remain an understudied group. The purpose of this study was to explore how girls with ADHD described their identity work amid the tension between norms of socially appropriate female behavior (conceptualized as the *Me*) and their true selves (conceptualized as the *I*). Ten participants aged 15 to 18 years were included. A thematic inductive analysis was conducted, complemented by an abductive approach incorporating a dramaturgical perspective and the concepts of *Me* and *I*, impression management, and social camouflaging. The findings broaden the understanding of identity work among girls with ADHD through four analytical themes: adjusting and suppressing behavior, navigating emotions and stereotypes, struggles in the school context, and the impact of medication. The results highlighted how participants struggled to conform to the roles expected by society, particularly within the school system. In their identity work, they also navigated the emotions that arose when confronted with the school’s idealized role model of a “female student.” Stereotypical thinking further extended to perceptions of the ADHD diagnosis. The school system played a significant role in shaping identities and influencing the participants’ experiences and self-perceptions. This process was reported to be both demanding and challenging. Schools often initiated the ADHD assessments. Following diagnosis, medication was prescribed, which, while offering some benefits from an educational perspective, also came at a cost. The price to be paid was an identity transformation—becoming someone other than who they truly were. This process infused their identity work with reflections on who they really were versus who they were expected to be in a society that places high value on academic achievement and performance. From a Swedish ADHD perspective, social factors—such as roles, norms, standards, and ideals—must be considered in relation to the rising number of diagnoses. There may be a tendency to quickly seek medical explanations for deviant behavior, particularly in school settings, which can profoundly impact identity work, especially for girls on the ADHD spectrum. Some solutions may lie beyond the medical approach and instead be sought in organizational, pedagogical, and resource-based changes. Considerably more research is needed on the understudied group of girls with ADHD to better understand their identity work and the role of both society and schools in the increasing prevalence of diagnoses.

## Introduction

Attention-Deficit/Hyperactivity Disorder (ADHD) is a common neurodevelopmental disorder, globally affecting approximately 5–7% of children and adolescents (Polanczyk et al., 2014). The number of individuals diagnosed with ADHD is increasing rapidly in Sweden, with an increase of almost 50 percent from 2019 to 2022 (Socialstyrelsen, 2023). Currently, roughly 10.5 percent of boys and 6 percent of girls aged 10–17 years have ADHD (Karolinska Institutet, 2024; Socialstyrelsen, 2025). The reasons underlying the increase are not fully understood, but proposed explanations include changing educational practices and policies, diagnostic routines, increased diagnoses among girls, and high acceptability of the diagnosis (Bölte et al., 2020; Hinshaw, 2018).

In Sweden, approximately two-thirds of children with ADHD are boys (Socialstyrelsen, 2025). However, in adolescence, this changes drastically, and among young adults, ADHD now appears to be slightly more common among women than among men (Socialstyrelsen, 2023). Therefore, it is urgent to study what ADHD means specifically for girls and their identity work.

Thus, the purpose of this article is to explore how girls with ADHD—a group that is understudied—describe their identity work amid the tension between generalized norms of socially appropriate behavior for females (conceptualized in this article as a role and the *Me*) and their true selves (conceptualized in this article as the *I*). The purpose of the article will be fulfilled through the analysis of 10 interviews with girls aged 15 to 18, using an inductive approach. The findings will then be abductively interpreted in the results section, informed by Goffman’s (1956) dramaturgical perspective on role-playing across different social and private stages, as well as Mead’s (1934/2015) concepts of the *I* and the *Me*. By doing so, this study aims to broaden the understanding of identity work among girls with ADHD.

### The inner workings and social dynamics of ADHD

The core symptoms of ADHD can manifest in different ways, but primarily involve difficulties with impulse control, attention, and hyperactivity (American Psychiatric Association, 2022). To be diagnosed with ADHD, an individual must exhibit symptoms in at least two areas of life, the symptoms must cause significant impairment or dysfunction, and the difficulties must have been present before the age of 12 (American Psychiatric Association, 2022). There are three subtypes of ADHD: the inattentive type, the hyperactive–impulsive type, and the combined type (American Psychiatric Association, 2022).

ADHD is associated with a higher risk of comorbidity with other psychiatric diagnoses, such as dyslexia, anxiety, and depression. ADHD treatment typically includes various forms of psychotherapy, as well as central nervous system (CNS) stimulant medication. In Sweden, 75 percent of children diagnosed with ADHD begin medication treatment after receiving their diagnosis (Karolinska Institutet, 2024; Socialstyrelsen, 2023).

Historically, ADHD has been considered a diagnosis primarily affecting boys (Faheem et al., 2022). While it is now well established that both boys and girls can have ADHD, boys are diagnosed earlier (Hinshaw et al., 2022; Socialstyrelsen, 2023) and a Swedish study found that girls made more visits to psychiatric clinics before receiving a diagnosis compared to boys (Klefsjö et al., 2020). Girls are initially often overlooked because their symptoms tend to be more internalized or misdiagnosed as anxiety or depression (Hinshaw et al., 2022; Klefsjö et al., 2020). Research suggests that girls develop ‘coping skills’ to mask or compensate for their behaviors, which leads to their ADHD going unnoticed (Klefsjö et al., 2020). As a result, it is more common for females to receive an ADHD diagnosis later in adulthood (Hinshaw et al., 2022; Socialstyrelsen, 2023).

ADHD is associated with temperamental traits (Nigg et al., 2004), making it closely intertwined with self-image and personality (Jones and Hesse, 2018). Individuals’ reactions to receiving an ADHD diagnosis vary and may depend on their prior identity and social connectedness. For instance, socially conforming youth may experience an identity conflict upon receiving an ADHD diagnosis, as the image of an unruly child is at odds with their self-image, whereas individuals with limited social lives may experience anxiety, because the diagnosis reinforces their experiences of not fitting in socially (Jones and Hesse, 2018).

There is limited research on how people with ADHD perceive, describe, and construct their identities. The diagnosis may be seen as something external, to distance oneself from Nielsen (2017) and Ringer (2019), but also something to identify with, a means of self-understanding, and to be proud of Andersson Frondelius et al. (2019) and Nielsen (2017). An ADHD diagnosis may create a separation within one’s identity, with an ambivalence between constantly adjusting to societal expectations and demands while simultaneously striving to accept oneself (Ringer, 2019). In a qualitative study by Nielsen (2017) ADHD was generally not described as an illness but rather as another way of being human.

A Swedish phenomenological study found that adolescents diagnosed with ADHD perceived their diagnosis as something that could accommodate their ADHD-related traits, which were described as both positive and negative (Andersson Frondelius et al., 2019). The participants made sense of their diagnosis by navigating both the strengths and challenges associated with it, both of which were considered essential parts of their identity. Social interactions with others who also had ADHD played an important role in shaping their understanding of and identification with the diagnosis (Andersson Frondelius et al., 2019). However, in interactions with new acquaintances who were unaware of their diagnosis, participants felt the need to disclose their ADHD to avoid being perceived as mentally ill (Andersson Frondelius et al., 2019). How girls with ADHD construct their identity is not well understood, which has been a key driving reason for this study.

### ADHD and school

ADHD is often identified in institutional environments such as schools (Rafalovich, 2013) and children and adolescents with ADHD face a high risk of experiencing both social and academic challenges in school (Tiikkaja and Tindberg, 2022), with increasing challenges across the school years (Johansson, 2021). Having friends at school and feeling safe in the school environment are key factors in fostering positive school experiences, yet these factors are often underrepresented among students with ADHD and other disabilities (Tiikkaja and Tindberg, 2022). The increase in ADHD diagnoses suggests that more individuals require healthcare and support, particularly in school. Yet, it has been suggested that ADHD symptoms remain relatively stable over time (Rydell et al., 2018; Willcutt et al., 2012). Thus, it is unlikely that the actual prevalence of the condition has increased. Instead, social factors—such as roles, norms, standards, and ideals; social context; reduced resources and reorganizations within schools; and stressed parents who want their children to thrive—may contribute to the rising number of diagnoses. Additionally, there may be a tendency to quickly seek medical explanations for deviant behavior, especially in school settings, which could also be a contributing factor. For children and adolescents, the school system plays a significant role in shaping their identities, as their experiences influence their understanding of themselves (Flum and Avi Kaplan, 2012; Gouvea, 2020; Hernández and Darling-Hammond, 2022; Verhoeven et al., 2019).

In Sweden, educational regulations state that all students, regardless of disability or formal diagnosis, should be able to attend school and have the right to support (Swedish Educational Act 2010:800, Chapter 3, §5). A formal diagnosis, such as ADHD, often leads to better access to special aids (Bölte et al., 2020) and adjusting the school environment by implementing smaller class sizes and providing social and pedagogical support is an important measure to support students with ADHD (Lindblom, 2023).

Stimulant medication is often regarded as an aid in schooling children with ADHD as it has been found to enhance concentration in learning environments (Prasad et al., 2013). However, the subjective experience is not necessarily well captured in these studies and a qualitative study found that while some participants experienced improved concentration, others felt that the medication altered their personalities, making them quieter and less spontaneous than they were naturally. Taking medication thus were regarded as a trade-off between academic achievement and personal authenticity, as participants felt they had to suppress aspects of their personality to succeed in school (Cooper and Shea, 1998).

Attending Swedish schools comes with specific behavioral expectations. Schools instill respect for authority, tolerance, and structure. Students are expected not only to complete their schoolwork and achieve passing grades but also to focus and concentrate, remain quiet, sit still for extended periods, and develop sufficient social competence to maintain friendships. In this way, school imposes behavioral demands that actively shape students. Individuals with ADHD often experience academic challenges and high rates of school absenteeism (Johansson, 2021, p. 164; Tiikkaja and Tindberg, 2022, p. 2). Beyond being a place of learning, school functions as a social institution that plays a central role in socialization and in the broader societal structure. It contributes to shaping students’ identities, including their knowledge base and core societal values (Gouvea, 2020; Hernández and Darling-Hammond, 2022; Verhoeven et al., 2019). Therefore, it is important to explore the experiences of girls with ADHD, and how these experiences influence their identity work.

## Analytical tools for studying identity work

### Identity work

Watson (2017) conceptualizes *identity work* as the ongoing processes through which individuals actively construct, negotiate, and present their identities within diverse social contexts. From this perspective, identity is not a stable or intrinsic characteristic but is continuously shaped and reshaped through interactions with social norms, cultural expectations, and organizational structures (Watson, 2017; Alvesson and Willmott, 2002). Rather than being fixed, identity is fluid, context-dependent, and subject to transformation as individuals navigate different social arenas (Sveningsson and Alvesson, 2003). Within working life, individuals often find themselves compelled to adjust their identities to align with professional roles or the prevailing culture of their organizations. This process is rarely straightforward; identity work frequently involves managing tensions between personal values and the demands imposed by social and institutional environments (Alvesson et al., 2008). It represents a delicate balancing act aimed at achieving both social legitimacy and the preservation of personal authenticity (Brown, 2015). Ultimately, identity work emerges as a dynamic, and at times conflictual, process through which individuals strive to produce a coherent and meaningful narrative of the self (Watson, 2017).

Identity work can be studied through people’s narratives about who they are and how they define themselves (McAdams et al., 2002, 2006; Mishler, 2004; Watson, 2017). This article explores how the participating girls describe the roles and identities they are ascribed, in relation to how they perceive themselves, and how this affects their identity work.

To analytically understand this identity work (Watson, 2017), a dramaturgical perspective based on Goffman’s (1956) work is applied, along with Mead’s (1934/2015) concept of the *Me* (society’s values that shape an identity within the self) and the *I* (the original part of the self).

### Conceptualizing performance from a dramaturgical role perspective

The dramaturgical perspective, as presented by Goffman (1956), describes how people use different types of masks to perform roles in various situations in order to conform to the group. Goffman also distinguishes between frontstage and backstage behavior. The frontstage is where we perform to fit in and gain approval from societal norms (Goffman, 1956). The backstage, in contrast, is the area where we can relax and be ourselves without adjusting to anyone else’s expectations. Goffman (1956) provides examples of backstage regions in the workplace, such as a personnel area or storeroom, where an individual can drop their performance intended for the audience, which, in this case, might be customers in a store. He points out that different languages are used for both the backstage and frontstage regions across society. The frontstage is where one must perform to meet societal expectations, while the backstage region “allows for minor acts that might be taken as symbolic of intimacy or disrespect for others present in the region” (Goffman, 1956, p. 78).

Moreover, people behave more formally in the frontstage, adhering to societal norms and expectations, while the backstage region allows for informal behavior, trusting that no audience is present.

People perform differently depending on the context—such as in the workplace, at a family dinner, or at a grocery store—because social expectations differ in each situation, and we take on different roles in each. In different contexts, we manage and adjust our behavior to influence how others perceive us. Goffman called this behavior “impression management” (Goffman, 1956, p. 132). Impression management is a vital part of social interaction, practiced by everyone in social settings, either consciously or unconsciously. It is not necessarily about what is morally good or bad, but rather how we wish to be perceived by the group to which we belong.

A well-known study that extended Goffman’s original concept of impression management and critically examined its impact on individuals’ emotional lives and well-being is Hochschild’s seminal research on flight attendants (Hochschild, 2003). Hochschild demonstrated that the requirement to continuously display friendliness and care was not merely about managing outward impressions but involved a deeper regulation of employees’ internal emotional states. Through the concept of emotional labor, she illuminated how emotional regulation became a commodified aspect of work, often resulting in emotional dissonance and psychological strain.

Social camouflage is a term closely related to Goffman’s (1956) role theory and impression management, though it focuses more specifically on hiding symptoms of psychiatric conditions and disabilities, primarily in autism research. Studies suggest that people with ADHD, especially girls, often engage in social camouflaging, which is linked to mental health challenges such as anxiety and depression (Kelly et al., 2024; McKinney et al., 2024; van der Putten et al., 2024). The term can be applied to ADHD as a form of impression management. Social camouflage occurs when an individual learns through socialization that expressing certain symptoms is undesirable. It involves actively mimicking others’ behaviors and can happen both consciously and unconsciously.

### Society’s norms as the *Me* that control the original *I*

Mead’s (1934/2015) concept of the generalized other refers to the values of society that become an integral part of our identity. The generalized other is developed through socialization and is so deeply embedded in us that we experience shame or guilt when we break the internalized norms (Trost and Levin, 2018). These norms, expectations, and societal scripts guide our behavior accordingly. Through the generalized other, an individual can understand the social consequences of their actions and perceive the perspectives and expectations of society. Holdsworth and Morgan (2007) describe the generalized other as an internal conversation with oneself, in which we take on the role of another person. They illustrated this by showing that participants in their study said things like “that’s what people do” or “people say to me,” revealing that the generalized other is used in everyday practices of evaluation and comparison (Holdsworth and Morgan, 2007, p. 414).

The generalized other shapes the part of the self, called the *Me*, which is developed and created in a social context. The *Me* helps the self to understand, interact with, and define situations in light of cultural symbols—particularly language, morals, values, meanings, and practices—that emerge from a specific social context (Mead, 1934/2015, p. 209). Mead distinguished between two parts of the self to fully understand it analytically: the *I* and the *Me*. In contrast to the *Me*, the *I* represents our uncontrolled, impulsive behavior, while the *Me* reflects the internalization of social norms and expectations. The *Me* acts as a regulator and even a censor for the *I* (Mead, 1934/2015, p. 204). Mead argues that social control sets limits on behavior and is an expression of the *Me*’s influence over the *I*. The *I* is the spontaneous, original part of the self, acting in the present moment (Trost and Levin, 2018), while the *Me* stores information and experiences and internalizes societal norms. Thus, the *Me* exercises social governance and control over the *I* (Mead, 1934/2015).

The concepts of the generalized other, *Me*, and *I* are analytical tools that help in understanding and describing the identity work of individuals. These concepts are particularly useful for analyzing, for example, female adolescents with ADHD, who must navigate their original experience of themselves in relation to a school-expected *Me* or mask/role, linking it to Goffman’s (1956) dramaturgical approach.

## Method

This study is part of a larger research project on ADHD and identity formation among adolescents with an ADHD diagnosis and those without a diagnosis but with symptoms. The research project has been approved by the Swedish Ethical Review Authority (reference number 2023-06162-01).

Since the aim of this study was to explore and describe girls’ experiences of their *identity work* (Alvesson and Willmott, 2002; Alvesson et al., 2008; Brown, 2015; Watson, 2017), a qualitative research interview method was applied (Kvale and Brinkmann, 2009). In-depth interviews are particularly useful when exploring participants’ experiences, feelings, and self-descriptions (Tjora, 2012).

### Sample and selection

This study was conducted as part of a larger research project on ADHD and identity (see, for example, Frick et al., 2025), which included both girls and boys who had received formal ADHD diagnoses as well as individuals who exhibited prominent symptoms but, for various reasons, had not been formally diagnosed. To specifically investigate the identity work of girls with ADHD, a targeted selection of 10 interview transcripts was made. These interviews were chosen based on their clear relevance to the study’s focus on ADHD and their explicit engagement with issues of identity work.

Ten interviews were deemed sufficient to achieve saturation in terms of experiences and diversity of perspectives. All participants were in upper secondary school or high school. Thus, the age span of the participants was 15–18 years. The participants lived in the greater Stockholm area at the time of the interviews.

### Interview methodology

The interviews were conducted by master’s students under the supervision of the project investigators, Author 1 (JG) and Author 3 (MF). The interview guide used in this project consisted primarily of open-ended questions, which provided ample room for subjectivity, interpretation, and flexibility. The interview guide specifically explored experiences related to home, school, and free time by asking questions and allowed for follow-up questions.

The interview guide is included in Appendix 1.

The interviews were collected during the spring of 2024 and were analyzed later that same year by Author 2 (ME), in collaboration with and under the supervision of Author 1 (JG). The use of interview data collected by our master’s students has both advantages and disadvantages. A clear disadvantage is that the material is “static,” meaning no follow-up questions can be asked. However, an advantage is that entirely new perspectives can be applied to the material, potentially uncovering novel insights. Since we had no direct contact with the participants, we presumably approached the material without developing participant-related preconceptions. All researchers inevitably bring their own perspectives and biases to an analysis, but in this case, such influences were likely minimized.

### Thematic inductive coding

A thematic inductive coding was employed to analyse the interview transcripts based on the process described by Braun and Clarke (2006). Thematic analysis is a well-established and flexible approach to qualitative research. This process provides clear guidelines for the method, while still allowing flexibility and the ability to move back and forth between the material during the analysis. An inductive approach is a bottom-up method, where the themes that emerge are closely related to the data itself (Braun and Clarke, 2006, p. 83).

The first step in the thematic analysis was to become familiar with the interview data. Author 2 (ME) read through the interview transcripts thoroughly multiple times, taking notes on segments that were relevant, followed by an initial coding of the material. This process sorted out the parts related to the focus of this study, i.e., identity work and school, and gathered them in a separate document.

The second step in the process was to look for themes within the codes, create themes, and review whether they fit. This was an iterative process, going back and forth to check the trustworthiness of the developing themes. The rationale behind this process is that themes should reflect the included codes, and vice versa. However, the rich nuances of the codes are somewhat lost in the overarching themes. Nevertheless, to present the coding in a meaningful way, the codes needed to be organized into broader themes.

The coding generated four overarching themes central to the participants’ identity work.

- *Adjusting and suppressing behavior,*
- *Navigating emotions and stereotypes,*
- *Struggles in the school context,*
- *Medicine’s impact.*

To ensure the rigour and credibility of the inductive process, the coding was conducted collaboratively by Author 2 (ME) and Author 1 (JG) during the autumn of 2024. The joint coding sessions allowed for continuous dialogue and reflexive discussions, contributing to a more nuanced and trustworthy interpretation of the data. JG held overall supervisory responsibility for the coding process, in close collaboration with Author 3 (MF), which further strengthened the consistency and coherence of the analysis. To enhance the transparency and replicability of the study, the coding scheme and detailed description of the coding process are provided in Appendix 2.

### The abductive-interpretative phase

Once the themes had been systematically distilled, the analytical concepts of Goffman (1956) and Mead (1934/2015) were employed to enrich and cross-fertilize the thematic coding. This process aligns with an abductive approach, which seeks to deepen and refine analytical interpretation (Vila-Henninger et al., 2024; Strauss and Corbin, 1990; Tavory and Timmermans, 2014). Abductive reasoning enables a higher level of analytical abstraction by integrating theoretical frameworks into the interpretation of thematic coding.

An abductive approach constitutes a methodological stance that combines elements of both induction and deduction but fundamentally seeks to generate new understandings through a dynamic, iterative movement between empirical data and theory. The abductive process aims to produce the most plausible and creative explanations for observed phenomena. In abductive analysis, researchers often begin with preliminary empirical material or a partially developed analysis. When this material is brought into dialogue with theoretical concepts, the process may lead to the refinement of existing concepts, the development of new ones, or the emergence of entirely novel perspectives (Strauss and Corbin, 1990; Tavory and Timmermans, 2014).

## Results

**Figure 1:**
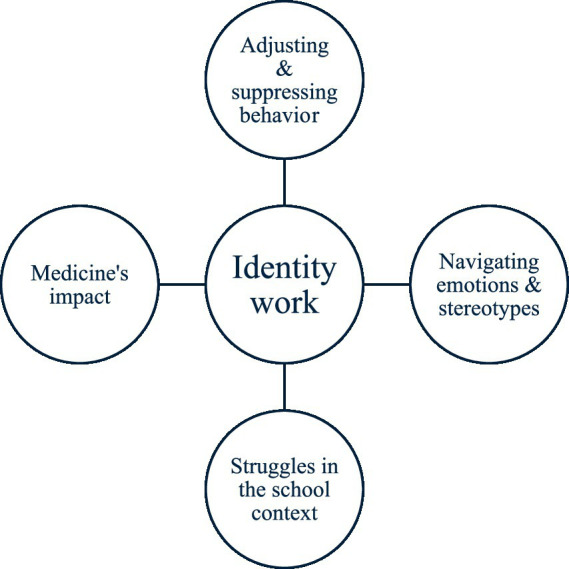
The identity work themes identified among the participants.

The results of the abductive interpretative phase are presented in this section. They are organized around four themes that were identified through inductive thematic coding as central to the participants’ identity work, as illustrated in Figure 1. For the sake of clarity, these themes also serve as the headings for the presentation of the results.

To ensure the anonymity of the participants’, identifying information has been omitted, and fictitious names have been assigned to each participant.

### Adjusting and suppressing behavior

A common theme was that the participants felt they had to adjust their behavior in different situations—mostly in the school setting but also in groups with new people—where it was unclear whether their behavior was appreciated. Multiple interviewees expressed that they had to hide their true selves and “hold back energy” to be socially accepted or to avoid being perceived as annoying by their peers. This is exemplified by quotes from Ester and Jasmine later in this section. However, this adjustment could also work in the opposite direction: participants who identified as introverts sometimes adapted their behavior in school to appear more outgoing and social, especially when there was a lot of “fooling around” in class.

Trying to fit into a group and adjusting one’s behavior to some extent is likely something all adolescents experience, as it is part of growing up and finding one’s place in the world. However, the experience for adolescents with ADHD may differ because they often learn that their traits or dispositions are considered inappropriate. Since ADHD entails hyperactivity and impulsivity, many participants expressed that they had to suppress those aspects of themselves. This process also involved observing others’ behavior and imitating it to some extent in order to fit into the group.

Hanna recounted:

*Well, one learns to adjust according to the environment you’re in and… for me it has resulted in… that maybe I become a lot quieter than I really am. Kind of diminishes my personality very much.*

Hanna continued to explain that if she relaxed in a social situation, she might say something inappropriate. Therefore, she remained tense and felt like she was “walking on eggshells” in contexts that did not feel entirely safe or familiar. She described this as very tiring, leading to a build-up of emotions that would later be expressed in a safer context.

Hanna explained:

*And that has often been the case for me, that when I come home from school or come home from an activity, I become entirely incapacitated, I just lay on the couch and take a breath and die just because… it wears you down, this social interaction.*

Another participant, Sara, had a similar experience of school being very draining. She was assessed for ADHD primarily because she was so exhausted.

Sara testified:

*I fell asleep every day after school directly when I came home, and I slept for like three hours because I was so exhausted from school.*

Camouflaging symptoms and constantly being on edge in social situations was described as necessary for functioning socially (McKinney et al., 2024; van der Putten et al., 2024). This behavior stemmed from a fear of saying something wrong or behaving in an undesirable way. Applying Goffman’s (1956) dramaturgical perspective, performing in the social front region was highly exhausting, creating a strong need for rest and recovery in a safer environment afterward. In line with Goffman (1956), impression management, as compellingly illustrated by Hochschild (2003), extended beyond the regulation of outward appearances to encompass the deeper management of participants’ internal emotional states, often leading to emotional and psychological exhaustion after school.

The safer context—often the home—functioned as the back region, where one did not have to perform or adjust their behavior but could simply be their original self, known as the *I* in Mead’s (1934/2015) framework. It should be noted that all people, regardless of diagnosis, adapt their behavior in social situations and require some degree of recovery in a back region. However, for individuals who actively suppress symptoms and constantly monitor their behavior, such as those with ADHD, this process is significantly more strenuous and therefore demands much more recovery time.

Ester described a similar experience:

*So, for example that I do not dare to have so much energy, because it feels like other people can find me annoying. So, I usually hold back a little bit and take a step back rather than being too intense.*

Ester explained that this had both negative and positive outcomes. The negative was that it was tough and required a lot of energy. The positive was that it prevented others from feeling uncomfortable due to hyperactivity and impulsivity.

The pros and cons of masking were also described by another participant, Anna. She explained that she tended to talk about topics she knew others were interested in while holding back her own interests and actively trying to fit in.

Anna recounted:

*It is both positive and negative I think, because I am not myself in the same way. But at the same time, it is fun to be able to have things in common to some extent.*

Camouflaging (McKinney et al., 2024; van der Putten et al., 2024) transformed one into someone other than their original, unfiltered self, yet it granted access to social communities where they could feel included. Applying Mead’s (1934/2015) theories, the *Me* controlled and restrained the *I*. The social role held significant power, compelling the *Me* to impose conformity upon the individual *I*, aligning their behavior with society’s internalized norms.

Thus, one could experience a sense of belonging and community, but at the cost of losing originality of the self. The ability to camouflage could also be considered an asset—part of a double-edged sword.

Another participant, Jasmine, described how she felt in certain social situations at school:

*Everyone is pretty calm, and I am very fired up and stuff and they think it is annoying so one must be calm and fit in and that I find that difficult.*

Multiple participants explained how they had taken on the role of “the class clown” and were often perceived as funny by their peers. Hanna explained that when she was younger, she used to feel left out and different at school. She assumed that others perceived her in a negative way, which led her to act out. As she grew older, she learned to use her hyperactive and impulsive traits, as well as her lack of a filter, to her advantage—turning them into useful tools in social situations.

Hanna explained:

*I mean, it became more appreciated that I don’t have much of a filter. It becomes more appreciated as I become older. I am bonding much better with adults because they think it’s funny with a teenager that can talk back. But still in an acceptable way. So, the older I’ve become, more people find it funny, and I guess I took advantage of it and maybe became the clown of my friend group.*

The older version of Hanna, the person she had become, had learned to balance the social expectations of *Me* and the impulsive *I* in such a way that *I* became a positive force in her life. It meant a lot to Hanna that the originality in her was appreciated by others as something positive.

Hanna testified:

*It is very important to me because I worry a lot that people find me weird. So when people instead find me funny, it makes me really, really happy and very thankful and it makes me feel that I can be myself and relax in a totally different way.*

Thus, a disposition that may be seen as undesirable in social contexts—such as in school, or before one has learned to control it socially—can actually be used to one’s advantage. Instead of being perceived as annoying and weird which deeply impacts the looking-glass self-perception, it can also make a person appear funny and spontaneous. This is a form of impression management (Goffman, 1956), in which Hanna and others adjusted their behavior to be perceived as spontaneous, direct, and funny.

### Navigating emotions and stereotypes

Most participants perceived their diagnosis as a significant part of their identity—who they were—which influenced how they functioned and processed information. The interview data revealed no signs of stigmatization regarding having ADHD per se; on the contrary, the participants stated that many people had an understanding of the condition. It was perceived as common and normalized by others. However, behaviors associated with ADHD were discouraged by certain individuals or in specific environments, such as in a school setting or at home.

Alma described how she identified with aspects of her ADHD and how difficult it felt when someone implicitly or explicitly suggested that the behaviors needed to be better controlled, for instance, with medication.

Alma testified:

*When someone points out the hyperactivity, like something negative. And that has not affected me before. Then it really has been like… it is a part of me, and I am proud of who I am. But recently, my mom has said things like, “I hope the medicine helps for the hyperactivity because it becomes very intense at home”, and I can understand that. It is just really difficult to hear.*

Receiving comments about the undesired characteristics that constituted the participants’ *I* inevitably generated substantial emotional labor that they were required to navigate (Hochschild, 2003). Confronting such feedback was genuinely emotionally taxing, further amplifying the intensity of their ongoing identity work.

Other participants explained that they struggled to identify with ADHD due to the strong stereotypes that exist about the condition. They did not see themselves in the way the diagnosis was portrayed by some people.

Hanna recounted:

*I do not even understand how I got diagnosed, because the questions they asked me were like “Can you sit still on a chair?” “Do you have to yell cuss words at your teachers?” I mean these were really strongly aimed questions. And then I felt like, this does not really apply to me. I am not like that, like I throw stuff and like… It feels like people with ADHD are described like we are crazy.*

Hanna problematized how ADHD manifested differently in boys and girls and how the common perception of ADHD was based on the stereotypical “boy ADHD,” which assumed that individuals with the condition were disruptive. She also pointed out that her ADHD went unnoticed for a long time, as the adults in her life simply saw her as “defiant.” Hanna understood this in relation to gender roles—girls were expected to behave in a certain way, and guilt was often imposed on them. Meanwhile, boys’ behavior was more accepted to a certain extent, giving them a better chance of having their struggles recognized and being diagnosed with ADHD early on. While awaiting proper attention and struggling with a different set of symptoms, girls were instead unable to see themselves as having ADHD due to the one-sided and widespread dissemination of a gender-coded boy stereotype.

Hanna explained:

*Then for the girl, for me as a girl, you think that “it is my fault that I act this way, that I think too much or that I am hyperactive or etcetera”. Because there is no way that I have ADHD, because I am not like them.*

According to previous research (Jones and Hesse, 2018), receiving an ADHD diagnosis is a unique experience for each individual but is likely to affect one’s identity. Many participants stated that receiving a diagnosis was a relief—it provided something to relate to and rely on, offering an explanation for why things did not work the same way for them as they did for their peers. However, it can also be an anxiety-inducing experience, as it places one in a social group to which society and the media have attached various meanings. Either way, the diagnosis generates identity work from new perspectives.

Tone described how the doctors discussed ADHD, informed her about the associated risks, and explained the possible side effects of the medication. She was told that she had a higher risk of depression and substance abuse, among other things. Receiving all this information placed her in a social category that was difficult to fully accept. In this case, the diagnosis was frustrating and disappointing, prompting identity work focused on concerns about the future.

Tone recounted:

*Because when I got this diagnosis, I was like, fuck, am I going to need medication to be able to sit still on a chair? What the fuck is that. It is not supposed to be that fucking hard.*

Receiving the diagnosis also meant a new definition of the situation and of who one was. Tone and other participants were labeled into a new group. At the same time, this involved new information for the *I*, which was now formally labeled as deviant by society— a form of deviance that needed to be treated with both medication and behavioral strategies so that the societal *Me* could better control and restrain the *I*.

Hanna described how she was often perceived as “stuck up” by others, and that she had no filter, which were not considered traditional feminine qualities. Going against society’s norms and expectations of how a girl should be also added an extra burden to the identity work. She simply wished that she could better match these socially ascribed traits.

Hanna recounted:

*So, there is absolutely a difference where I would like to be softer in my ways and well… more humble, nice and kind and maybe a little bit more what fits into the girl stereotype than I am.*

Ester experienced her ADHD symptoms as obstacles, particularly in school, where she struggled with concentration. However, she also emphasized that she was proud of her diagnosis and that the traits associated with it are a significant part of her identity.

Ester testified:

*I think my patience is not as good as others and I think that is because of my diagnosis. But in a way, it makes me who I am so I would not want to change that. Even though it would be nice to have some patience sometimes. But I would not want to change that because then I would not be me.*

Ester acknowledged the challenging aspects of her ADHD but still recognized that it was a fundamental part of who she was. Navigating and managing identity work in relation to ADHD was difficult. Many participants were proud of their diagnosis and the traits that came with it. However, it also led to confusion and frustration when others disapproved of it or when ADHD was understood in a stereotypical way that was hard to relate to. Additionally, society constantly signaled that such behavior was undesirable. As a result, the societal *Me* was continually trying to order, control, and restrain the *I*.

### Struggles in the school context

School was often challenging for the participants, especially when proper accommodations were not provided. All of them experienced difficulties in school to varying degrees. A distinction could be made between issues related to schoolwork (academic issues) and those concerning social interactions with teachers and classmates (social issues).

Participants reported difficulties with concentration and focus on school assignments, as well as challenges with executive functioning when starting a project. They had to manage an inconsistent ability to be productive—some days, they struggled to accomplish anything, while on other days, they could write several pages or complete multiple assignments.

The social challenges often stemmed from feeling left out, misunderstood, or overwhelmed by large classes, which intensified the identity work.

Another common issue was that typical ADHD symptoms—such as hyperactivity and impulsivity—often conflicted with the social norms and expectations of school. A specific *Me* was constructed within the school context: a student who was responsible, quiet, obedient to authority, and able to sit still. Many participants stated that they struggled with sitting still, staying quiet, and maintaining focus for long periods. As a result, the school *Me*’s effort to control the *I* was an exhausting process.

All participants in this study had been diagnosed with ADHD, and in almost every case, the initiative for a clinical assessment came from the school. However, the participants were not assessed until late adolescence because there were no visible problems in school or with their grades. Yet, it was often in school that it became clear they had ADHD or that ADHD first became an obstacle.

This means that, depending on the severity of the ADHD, it may not pose difficulties outside the school context.

Chrissy recounted:

*I think the biggest problem for me is that I don’t function in school. I mean outside of school; I don’t care about ADHD. Then I feel good, living my life but in school and when I work and that kind of stuff. It often becomes very hard and complicated […]*

Tone testified:

*My teachers at that school said that this is not working out, she has some kind of problem, so we must assess her for ADHD because she does not work, it does not work.*

Using Goffman’s (1956) dramaturgical perspective, the performance in the front region was extremely tiring and required a lot of recovery in the backstage region. The specific performance in the school environment involved maintaining a mask to play the role required at school. But it consumed so much energy.

Previous research on adolescents’ school experiences suggests that while the early years of schooling were relatively manageable, secondary and upper secondary school proved more challenging (Johansson, 2021). Several participants echoed this pattern, describing how the early school years were more accommodating of certain ADHD traits that later became less tolerated. Furthermore, the experience of having ADHD in school varies depending on the specific school context.

Chrissy thought her behavior was normal in the earlier years of school. Back then, she lived in a non-academic town. She described a behavioral and academic change later in middle school.

Chrissy recounted:

*When I got up to middle school it was more like you should be able to sit still. You should, it is expected by the teachers. You are supposed to manage this, the national tests became thrice as difficult.*

In fifth grade, she switched to a more academic and high-achieving school, where students were expected to sit still and listen. She described how the classroom became chaotic because of her behavior. A school *Me* is constructed by society, more or less prevalent in certain areas, and often becomes clearer after the first few years of school. These examples show the dependence on social context: in some environments, ADHD symptoms may not be seen as different or deviant at all, while in others, clear lines are drawn as to which behaviors are desirable and which are not. What is considered deviant or undesirable is largely socially constructed and becomes clearer in comparison to other groups.

Jasmine testified to having similar experiences:

*It worked out fine in elementary school. It was fairly easy. Then it became a little harder in middle school, I was pretty messy. Then I started upper secondary school and then I stopped going all together because I thought it was difficult, and I did not get any help.*

For Tone, school and the role of a student had almost always been associated with anxiety. She explained how she never felt comfortable in school, partly because she saw others perform in the classroom in ways that she was unable to.

Tone recounted:

*I mean, what is wrong with me? And it does not get better by adults imposing the idea that what I am doing will not work for the rest of my life and when you are a kid, you are like, oh. When you hear that kind of stuff, you are like, okay. So, I am supposed to be like this for the rest of my life and that does not work out.*

As the quote shows, adults were assigning a definite label to her, which created worry about the future. Using Mead’s (1934/2015) terminology, *the generalized other*—consisting of internalized societal values—was always present and constantly reinforced by the adults, who repeatedly emphasized what was expected of Tone and others. Demanding identity work was triggered when the self failed to meet the expectations of *the generalized other*, particularly in the constant comparison to others’ performances on the stage of the classroom.

The level of accommodations and support available to individuals with ADHD varies between schools. Ester described her school experience as a square, while she felt like a triangle, as she did not feel like she fit in. Moreover, she explained that although she actually liked school, the lack of accommodations and support generated negative feelings towards it.

Ella described how she usually has a good self-image, but that it had been damaged by how she was treated by teachers and friends at school, which speaks to a different *Me* or identity that felt really bad.

Applying Mead’s (1934/2015) concepts of *I* and *Me*, the expectations and norms within the school context became internalized, forming a school-specific *Me*. This school *Me* constrained the *I*, enabling individuals to function socially within the educational environment. This process can also be understood as a form of social camouflaging and impression management—actively controlling symptoms and expressions to conform. The analysis suggests that adolescent girls with ADHD must adopt a distinct dramaturgical role to fit in on the school stage (Goffman, 1956). This role, however, generated tension and friction, resulting in struggles with identity work and authentic self-expression in school.

### Medicine’s impact

The participants took some form of central stimulant medication. The attitude toward the medication varied, but the main reason for taking it was to increase focus and concentration in school. Thus, performing on the school stage served as the primary motive. Based on the analysis, the medication seemed to create two distinct identities and was often described as almost creating two different versions of themselves.

Chrissy testified:

*I am not myself when I am on the medication. And that is the reason why I do not want to take it. So, I am pretty much avoiding it. Which is not good, because then it is going like crap in school.*

Chrissy also explained how the medication helped with concentration in school and improved her grades. However, she stressed how it felt to take the medication since she did not feel like herself when she was on it. Teachers and parents wanted her to continue taking the medication so she could succeed in school, but her friends did not like the person she became while on the medication.

Chrissy recounted:

*No one of my friends wants me to take the pill because I am not myself and that also had an impact. Because they like me when I have ADHD. No one else does. My teachers do not, my parents. They want me to manage school while all the other people around me are like, you are so boring when you are on the pill, you cannot even take a joke.*

This created a conflict between two roles or identities. The medication created a *Me* fit for the school stage, which affected the *I* to such an extent that friends barely recognized her. The medication reorganized the self, and on the front stage at school, the calm and capable student *Me* was present. However, lost in the private social stage was the authentic, funny friend tailored to the *I*. This resulted in a conflicting situation involving several social and personal layers: teachers, parents, friends, and Chrissy.

Sara had a somewhat different experience over time. The primary purpose of the medication was to improve her grades in school.

Sara recounted:

*I tried Ritalin for a while. Then we increased the dose, and we continued to increase because I did not feel any difference and then I started to get a little headache. In first year of high school, I felt like, my grades are good, and I have not used the medicine for a while. Because, if I’m being honest, I forgot to take them. And I just felt like, no but if I can receive good grades without the medicine, then it is fine.*

Sara’s case speaks to the possibility, depending on the severity of the symptoms, of learning the role of a student and developing skills that can eventually be maintained without medication.

Ester, on the other hand, explained how she was trying different medications with varying doses to find the right one. The medication was helping her, but the most recent one stopped working in the middle of the last class. This may point to the possibility of change, even evolution, as articulated by Sara, and suggests that other factors may be at play over the many years spent in the school context among children and adolescents.

## Discussion

The analysis clearly illustrates that the *identity work* (Alvesson and Willmott, 2002; Alvesson et al., 2008; Brown, 2015; Watson, 2017) among girls with ADHD was a demanding process. Adjusting and suppressing behavior suggests the need to conceal ADHD traits to function in the school setting and to feel included and accepted in certain social contexts. It was an especially exhausting task to perform and devote to impression management on the front stage of the school context, as seen through Goffman’s (1956) dramaturgical lens. The findings align with Hochschild’s (2003) emotional and psychological insights on impression management, revealing how an entirely different group was similarly subjected to pressures to conform to a social and professional *Me* that, at times, conflicted with the selves they aspired to be and authentically embody.

The participants were subjected to imposed identity norms and behaviors by institutions such as the school (Flum and Avi Kaplan, 2012; Gouvea, 2020; Hernández and Darling-Hammond, 2022; Verhoeven et al., 2019), which sought to restrain and control their true self, or *I*, to use Mead’s (1934/2015) concept. This was a taxing effort that required extensive recovery afterward and was perceived as an intrusion on their authentic selves, creating a stream of thoughts and emotions. Navigating and handling these experiences in their identity work was demanding.

Similarly, it was also challenging to combat what the participants perceived as a prevailing gender stereotype of ADHD, which had various implications for them—ranging from a delayed recognition of their difficulties to postponed diagnoses and encounters with widespread stereotypical thinking about the condition (Faheem et al., 2022; Klefsjö et al., 2020; Hinshaw et al., 2022). Given that a significant portion of their lives revolved around school, it can be said that school had a profound impact on their identity work (Lindblom, 2023; Johansson, 2021; Rafalovich, 2013; Tiikkaja and Tindberg, 2022).

School was described as anxiety-inducing and challenging by most of the participants, who experienced various difficulties both academically and socially (Tiikkaja and Tindberg, 2022). The experience of not living up to expected academic and social performance standards contributed to a negative self-image and self-condemnation. This range of experiences, which is connected to performance, results, and assessment, also has a value component.

In other words, there was challenges related to the personal experience of academic and social worth—when one struggles to fit in and function both academically and socially—especially in a school context that fully gravitates toward performance, results, and grading (Lindblom, 2023; Johansson, 2021). The value aspect may particularly need to be addressed in the school environment regarding the identity work of girls and also boys with ADHD.

The idea of medication stemmed from society’s high regard for the ability to perform and deliver results in school (Cooper and Shea, 1998). Medication was seen as a necessary means to fulfill the role of a student. In this context, simply being oneself was not enough, a point that requires further discussion.

### Medication as an imposed means to play the role of a student in line with the expectations

School was generally the agent that initiated an ADHD assessment (Rafalovich, 2013). Teachers often initiated the assessment due to undesirable behavior, such as inattentiveness or hyperactivity, or low grades. However, for participants who had few difficulties in school and received good grades, being assessed for ADHD was more challenging. This suggests that initiating an assessment was more difficult when participants demonstrated strong academic performance, and that the process of being assessed posed a greater personal and identity-related challenge for those who succeeded within the school system’s normative frameworks. Thus, in order to receive an assessment, problems had to be visible or clearly interfere with schoolwork, which is not always the case for girls with ADHD (Klefsjö et al., 2020; Hinshaw et al., 2022).

Regardless of performance and results, school was the biggest obstacle and challenge for the participants. Some even stopped attending school in upper secondary education. This resonates with previous research, which shows that students with ADHD are more likely to be long-term absent from school (Johansson, 2021; Tiikkaja and Tindberg, 2022).

According to school regulations in Sweden, all students, regardless of diagnosis, have the right to special support and accommodations in school if needed (Skollagen, 2010). Almost all participants in the study had some form of special support but felt that it was insufficient.

In the end, school was experienced as a highly structured and rigid environment with little tolerance for behavior that deviated from norms and expectations. Additionally, there was an accelerating curve of increasing demands, as school was perceived to become more challenging after elementary school, reaching its peak difficulty in upper secondary school. This aligns with previous research that has shown the same pattern (Johansson, 2021).

Thus, the role of medication served as an imposed means to fulfill the failed role of a student. Many participants described how medication helped them, particularly with executive function difficulties. It contributed to better grades, made it easier to start projects, and improved focus in class, for example. Medication was often described in terms of “helping with grades,” “getting the job done,” and the importance of it lasting throughout the entire school day. Most participants started medicating soon after receiving their diagnosis (cf. Karolinska Institutet, 2024).

Not all participants reported issues related to their medication, and it is important to note that the role of medication is neither inherently good nor bad but rather complex (Bradby, 2016; Cooper and Shea, 1998). The analysis suggests that medication was used almost exclusively to enhance academic performance, which is a perspective worth problematizing from an identity perspective.

Central stimulant medication also comes with several side effects, both physical and emotional, which distinguishes it from other types of medication as it significantly affects one’s personality and behavior (Bradby, 2016; Cooper and Shea, 1998). Participants in this study, as well as previous research (Cooper and Shea, 1998; Nielsen, 2017), have shown that this can create ambivalent feelings about one’s identity. Medication clearly influenced their identity work and fueled questions about their different identities and, implicitly or in some cases explicitly, about their self-worth—both in school, at home, and elsewhere. *The generalized other* (Mead, 1934/2015) was thus clear in its communications and interventions—particularly through identity- and emotion-altering medication—that the role of a high-achieving student with good grades took precedence over the *I* with ADHD who struggled in school. The questions they faced in their identity work were left for the participants to navigate and resolve on their own.

Moreover, identity-altering medication is not necessarily the sole solution from a school perspective for adolescents with ADHD. This can also be viewed from an organizational and resource perspective (Lindblom, 2023; Johansson, 2021). Given the fact that more students struggle to manage school to the extent that they require medical interventions, alternative solutions may lie in how education is organized.

From both a pedagogical and a cost perspective (rather than relying on expensive assessments, medication, and specially designed support functions), significantly smaller class sizes with qualified educators could be another approach to supporting the growing group of students with ADHD (Lindblom, 2023; Johansson, 2021). In fact, a current public debate in Sweden (Bengtsson, 2025; SNPF, 2024) and recent research (Folkhälsomyndigheten, 2023; Högberg et al., 2019) have highlighted this issue, suggesting that the Swedish school system is structured in a way that many students find stressful and difficult to manage and navigate. New, old, or entirely different ways of organizing education need to be considered as an alternative to medication, with significant consequences for identity work, self-understanding, and the importance of being allowed to be oneself.

Another approach could be to reconsider early national tests, which start in year 3, and the grading system, which currently begins in sixth grade in Sweden. Taken together, these factors contribute to high expectations and demands being placed on children at a very early stage. Research is inconclusive regarding these changes to the school system and has not fully considered their impact on identity work and formation in children and adolescents.

### One-sidedness in the dramaturgical directing of a student role: maybe more stages are needed

Impression management (Goffman, 1956) in the school setting, which involves playing a very different role and suppressing a large part of one’s authentic *I* on a daily basis, creates confusion about one’s identity (Jones and Hesse, 2018). Adolescents with ADHD have learned that their behavior is often considered inappropriate or undesirable. While many participants were proud of who they truly were, society constantly reminded them that their behavior was problematic (Andersson Frondelius et al., 2019). Teachers or parents spoke to the participants about how their behavior was unacceptable in school and would not be beneficial in adult life. Once again, this generated significant *emotional labor* (Hochschild, 2003) that became embedded in their ongoing identity work.

Revisiting the studies by Ringer (2019) and Andersson Frondelius et al. (2019), which highlighted the ambivalence experienced by individuals with ADHD between adapting their behavior to meet societal expectations and embracing their ADHD traits, the participants in this study shared a similar struggle. They identified with and felt comfortable with their ADHD but often found themselves in situations where they felt the need to hide their traits. The participants described masking and adjusting their ADHD traits as exhausting.

Previous research on social camouflaging has shown that it is linked to poor mental health (McKinney et al., 2024; van der Putten et al., 2024). This suggests that creating inclusive environments that accept and accommodate their traits is crucial for this group.

Another approach, linking the challenges to the current organization of the school, could be to create more educational stages at the school. Instead of a large stage with a standard role, organizing more, but smaller stages would create new and better conditions for students. This approach would benefit both girls and boys with ADHD, reducing tension, turbulence, and conflict in identity work while improving conditions for mental health and well-being.

The interaction between the school system and the increased prevalence of ADHD diagnoses among children and adolescents is something that requires much more research. From a sociological perspective, it is not only medicine that has solutions for how the best possible conditions for students with ADHD are created. On the contrary, it is important to look beyond a medical paradigm to identify more sustainable paths from a long-term self-identity perspective (Bradby, 2016).

### Reflections, limitations, and future research

The theoretical perspectives used in this study have been highly valuable from an analytical standpoint, helping to understand the roles, or *Mes*, that society deems desirable and how these can contrast with what feels inherent and authentic to the individual. Among the participants, it is especially evident that they played a role on the school stage for much of their time, creating significant challenges for their identity work. However, our approach to abductive reasoning in the analysis has its limitations. For example, a more dialogical perspective could better account for the collaboration between the *Mes* and the *I*, such as by incorporating dialogical self theory (Hermans, 2001a, 2001b).

This study used a limited sample of 10 interview transcripts from participating girls with ADHD. Since this was a qualitative study, the aim was not to generalize the findings to the broader population, but rather to explore and gain a deeper understanding of the identity work of girls with ADHD, as this remains an understudied group. However, an obvious limitation of this approach was that no comparisons could be made between boys and girls. Such comparisons are intended to be conducted in the later stages of the project. Another limitation was that we analyzed previously collected material, which was “silent,” meaning no follow-up questions could be posed.

More research is warranted on the rising prevalence of ADHD among adolescents, taking into account the complex interaction between schools, parents, healthcare, and the individual. Additionally, the organization and resources of schools are important areas of research. Fundamental changes in schools—such as grading systems, higher expectations, large class sizes, a lack of pedagogical resources, and increased competition in higher education and the labor market—are likely to influence the focus on behavior, achievements, and performance. This, in turn, may lead to children being labeled early on within the ADHD spectrum, shaping their identity.

A longitudinal research design would have been a desirable approach. While such studies are undeniably resource-intensive, they hold the potential to generate unique insights into identity work both prior to diagnosis and at various stages following it. This would offer a process-oriented understanding of ADHD, the development of targeted interventions, and the evolving nature of identity work over time.

## Data Availability

The original contributions presented in the study are included in the article/Supplementary material, further inquiries can be directed to the corresponding author.
