# Supplementary material for: Identity work among girls with ADHD: struggling with Me and I, impression management, and social camouflaging in school
Source: Front Psychol. 2025 Jul 16;16:1591135. doi: 10.3389/fpsyg.2025.1591135 (PMC12308699; doi:10.3389/fpsyg.2025.1591135)
Supplement: Supplementary file 1 [file Table_1.docx]

**Appendix 1**

**Interview Guide**

**Living with ADHD: Identity Formation Among Adolescents and Young Adults with ADHD**

**Version 1: For Participants with an ADHD Diagnosis**

**Introduction**

• **Introduce yourself**
State your name, who you are (PhD student/master's student/research assistant), and engage in some general small talk to build rapport and make the participant feel comfortable.

• **Explain the purpose of the interview**
*"Today, I will interview you about your experiences and your perspective on what it has been like for you to live with your ADHD diagnosis."*

*"We are conducting this research to understand how adolescents and young adults experience living with ADHD, so that society can provide the best possible care and support for young people with ADHD. That’s why we really appreciate you taking part and sharing your experiences with us!"*

*"Participation in this interview is completely voluntary. If there are any questions you don’t want to answer, that’s absolutely fine. You have the right to stop the interview at any time without providing a reason, and you can withdraw from the study at any point."*

*"I will be recording our conversation, and later, the interview will be transcribed into text. The audio and text files will be securely stored to ensure that no unauthorized persons can access them. The results will be presented in a master's thesis and in scientific articles. No personal information will be disclosed, so no one will be able to identify you based on what you share."*

*"This interview will take approximately 1 to 1.5 hours. If you need a break, just let me know. As a thank you for participating, you will receive a gift card."*

*"Do you have any questions? If not, let's get started!"*

[Ensure that digital/written consent has been obtained before starting the interview.]

**Basic Questions Exploring Narrative Identity from a Present Perspective**

1. Could you start by telling me a little about yourself—who you are and what you enjoy doing in your free time?
2. Why do you think you are the way you are, and why do you enjoy these particular activities?
3. If you reflect for a moment, is there a difference between who you perceive yourself to be and who you would like to be?

**Basic Questions Exploring Narrative Identity in Relation to ADHD**

1. You have an ADHD diagnosis. Can you tell me about your experience of receiving the diagnosis (how old you were, why the assessment was conducted, what you thought beforehand, what changed afterward, how it felt to get the diagnosis, whether you told your school/friends, how it affected your self-perception/how others saw you)?
2. Have you received any special support at school or any treatment from child and adolescent psychiatry (BUP) due to your ADHD? Would you like to share more about these experiences?
3. Is there anything that could have been done differently?

**More Specific Questions Exploring Identity Construction in Relation to School**

1. How have you experienced your time in school? If it helps, you can divide it into different periods (e.g., elementary school, middle school, high school, university/college, etc.).
2. During your school years, have you ever felt like you became someone other than who you want to be or perceive yourself to be?
3. Would you like to share more about that?
4. How have you managed this conflict or gap?
5. What do you think could have been done to prevent this?
6. In what ways has this affected your relationships with classmates and teachers?

**More Specific Questions Exploring Identity Construction in Relation to Work**

(For participants whose primary occupation is employment)

1. How have you experienced your working life? If it helps, you can divide it into different jobs you have had.
2. Have you ever felt like you became someone other than who you want to be or perceive yourself to be at work?
3. Would you like to share more about that?
4. How have you managed this conflict or gap?
5. What do you think could have been done to prevent this?
6. In what ways has this affected your relationships with coworkers and supervisors?

**More Specific Questions Exploring Identity Construction in Relation to Leisure Activities**

1. How have you experienced your free time and any leisure activities? If it helps, you can divide it into different periods or activities you have participated in.
2. Have you ever felt like you became someone other than who you want to be or perceive yourself to be during your free time or leisure activities?
3. Would you like to share more about that?
4. How have you managed this conflict or gap?
5. What do you think could have been done to prevent this?
6. Are there any settings where you have felt that you could be exactly who you are or become who you want to be?
7. Would you like to share more about this?

**More Specific Questions Exploring the Influence of Healthcare on Identity Construction**

1. How has your contact with child and adolescent psychiatry (BUP) or other psychiatric services been?
2. Would you like to share how your contact with BUP has affected you?
3. Is there anything that could have been done differently?

**More Specific Questions Exploring Identity Construction in Relation to Others**

1. How have your relationships with others been? If it helps, you can divide it into different periods (e.g., early years, middle school, high school, university/college, etc.).
2. Have you ever felt that, in relation to others, you became someone other than who you want to be or perceive yourself to be?
3. Would you like to share more about that?
4. How have you managed this conflict or gap?
5. What do you think could have been done to prevent this?
6. Are there any other situations where you have experienced similar things? If so, please tell me about them.
7. How have you managed this conflict or gap?
8. What do you think could have been done to prevent this?
9. Are there any relationships where you have felt that you could be exactly who you are or become who you want to be?
10. Would you like to share more about this?

**Closing Questions**

1. Is there a question I should have asked to better understand you?
2. Is there a question you would like to revisit before we finish?
