# Supplementary material for: Identity work among girls with ADHD: struggling with Me and I, impression management, and social camouflaging in school
Source: Front Psychol. 2025 Jul 16;16:1591135. doi: 10.3389/fpsyg.2025.1591135 (PMC12308699; doi:10.3389/fpsyg.2025.1591135)
Supplement: Supplementary file 2 [file Table_2.docx]

**Appendix 2**

The coding process is illustrated by this coding scheme. It shows the process from quote to code to emerging theme, and includes a brief explanation of how each theme is defined. The quotes are connected to the codes next to them in the table.

**Coding scheme**

| Quotes | Code | Theme | Definition |
| --- | --- | --- | --- |
| ”for example, I do not dare having so much energy, because then it feels like others can find me annoying”  ”to not show that I have a diagnosis so I am trying to copy them how they behave”  ”for me it has resulted in that maybe I become a lot more quiet than I actually am. Kind of diminishes my personality pretty much” | Holding back energy to be approved  Copying others and avoiding showing signs of diagnosis  Hiding one’s personality, being more quiet | Adjusting and suppressing behaviour | Suppressing traits of ADHD in certain social situations as well as mimicking others to fit in |
| ”I am a typical ADHD kid. So I am loaded with 90% ADHD”  “I think my patience is not as good as others and I think that is because of my diagnosis. But in a way, it makes me who I am so I would not want to change that.”  ”Then for the girl, for me as a girl, you think that “it is my fault that I act this way, that I think too much or that I am hyperactive or etcetera”. Because there is no way that I have ADHD, because I am not like them” | Identifying oneself with the diagnosis: social identity  Conflict in identity, proud of ADHD traits even though they can be difficult  Guilt is put upon girls with ADHD – hard to relate to the male stereotype of ADHD | The complexity of identifying with ADHD | Complex relationship with ADHD: it is a large part of the identity, while other aspects are difficult to relate to or identify with |
| ”I think we started my assessment in seventh or eighth grade because my grades were really bad in school”  “Then I started upper secondary school and then I stopped going completely because it was so hard, and I did not get any help” | Assessment initiated by school  Lack of support in school | Struggles in the school environment | Adolescents with ADHD usually struggle both academically and socially in school. School is often the argument to do an assessment or start medicating. |
| “[…] I like to do in my free time what I have energy for..”  “You’re going to have to quit a social life if you want to manage school”  ”I attended a lot of sports and activities, but have always quit because I think it becomes boring or exhausting”  “I have always liked being creative and I have always liked drawing and painting and such” | Activities determined by energy  Social life and school not compatible  Appreciates calm and creative activities | The unattainability of free time | Free time is described as not compatible with school, since school takes all energy. Many respondents prefer unstructured, creative activities rather than structured free time activities. |

The coding process was supervised by JG.
